# Structural inequities in seasonal influenza vaccination rates

**DOI:** 10.1186/s12889-021-11179-9

**Published:** 2021-06-17

**Authors:** Lara I. Brewer, Mark J. Ommerborn, Augustina Le Nguyen, Cheryl R. Clark

**Affiliations:** 1 Center for Community Health and Health Equity, Brigham and Women’s Hospital, 1620 Tremont St., 3rd floor, Boston, MA 02120 USA; 2 Division of General Internal Medicine & Primary Care, Brigham and Women’s Hospital, 1620 Tremont St., 3rd Floor, Boston, MA 02120 USA

**Keywords:** Influenza, Health equity, Racial disparities

## Abstract

**Background:**

Influenza immunization is a highly effective method of reducing illness, hospitalization and mortality from this disease. However, influenza vaccination rates in the U.S. remain below public health targets and persistent structural inequities reduce the likelihood that Black, American Indian and Alaska Native, Latina/o, Asian groups, and populations of low socioeconomic status will receive the influenza vaccine.

**Methods:**

We analyzed correlates of influenza vaccination rates using the 2019 Behavioral Risk Factor Surveillance System (BRFSS) in the year 2020. Our analysis compared influenza vaccination as the outcome of interest with the variables age, sex, race, education, income, geographic location, health insurance status, access to primary care, history of delaying care due to cost, and comorbidities such as: asthma, cardiovascular disease, hypertension, body mass index, cancer and diabetes.

**Results:**

Non-Hispanic White (46.5%) and Asian (44.1%) participants are more likely to receive the influenza vaccine compared to Non-Hispanic Black (36.7%), Hispanic (33.9%), American Indian/Alaskan Native (36.6%), and Native Hawaiian/Other Pacific Islander (37.9%) participants. We found persistent structural inequities that predict influenza vaccination, within and across racial and ethnic groups, including not having health insurance [OR: 0.51 (0.47–0.55)], not having regular access to primary care [OR: 0.50 (0.48–0.52)], and the need to delay medical care due to cost [OR: 0.75 (0.71–0.79)].

**Conclusion:**

As COVID-19 vaccination efforts evolve, it is important for physicians and policymakers to identify the structural impediments to equitable U.S. influenza vaccination so that future vaccination campaigns are not impeded by these barriers to immunization.

**Supplementary Information:**

The online version contains supplementary material available at 10.1186/s12889-021-11179-9.

## Background

The Centers for Disease Control and Prevention (CDC) recommends annual influenza vaccination for everyone 6 months and older. The CDC estimates that in the 2018–2019 United States influenza season, 35.5 million individuals were sick with influenza, resulting in 490,600 hospitalization and 34,200 deaths [1]. Immunization against influenza is an excellent low-cost, safe, and effective way to reduce influenza morbidity and mortality. Unfortunately, influenza immunization rates are below public health targets throughout the U.S. population, and reflect persistent structural inequities that reduce the likelihood that Black, American Indian and Alaska Native, Latina/o, Asian groups, and populations of low socioeconomic status receive the influenza vaccine [2]. The U.S. does not currently provide federal funding for flu vaccination campaigns, although some states offer programs for lower-income patients that cover access to care—the result is uneven access to healthcare, which is a structural issue in the U.S.

Past literature has highlighted the impact of factors such as insurance coverage [3], accessibility of flu vaccine [4], education level [5, 6], income [6], employment status [5], housing segregation [7], and rurality on racial and ethnic disparities in influenza immunization rates in the U.S. [8] The persistence of these associations with vaccination rates reflects structural inequities and structural racism that should be addressed to reduce inequities in influenza mortality.

Structural racism impacts individual and population health through domains including, but not limited to unequal access to and provision of medical care [9]. The National Academy of Medicine’s compendium, “Unequal Treatment” describes several specific structural factors that contribute to disparities in care between racial and ethnic groups in the U.S., including fragmentation of healthcare systems that leave Black, Indigenous, Latina/o and Asian groups uninsured, or placed within lower-cost health plans that may lead to less access to primary care clinicians, and a lower ability to afford care due to cost [10]. The World Health Organization (WHO) frames the persistence of racial and ethnic disparities in access to clinicians, inadequate payment for care and delayed care due to cost as structural inequities that represent remediable choices within health systems, with deleterious consequences for marginalized groups [11].

In this analysis, we use data from the 2019 Behavioral Risk Factor Surveillance System (BRFSS) survey to examine racial and ethnic inequities in influenza vaccination uptake, and to estimate the contribution of three specific structural factors, insurance status, having a primary care doctor, and delayed care due to cost to differences in influenza vaccination uptake within and across racial/ethnic groups in the U.S.

## Methods

### Data

We examined data from the 2019 BRFSS [12]. The BRFSS is an annual cross-sectional random digit-dialed telephone survey of the United States civilian population aged 18 years and older which asks about health conditions and behaviors. The survey is administered annually by the CDC and state health departments in each of the 50 United States, the District of Columbia and select U.S. territories. The CDC uses raking weights to produce population estimates that adjust for survey non-coverage, non-response, and the probability of being sampled given the geographic location, age, race, and sex of the participant [13].

### Variables

Our outcome of interest was influenza vaccination in the past 12 months, which was assessed via the survey question, “During the past 12 months, have you had either a flu vaccine that was sprayed into your nose or injected into your arm?”

Age and binary sex were assessed via survey questions. Race/ethnicity was self-reported in the following categories: non-Hispanic White (NH-White), non-Hispanic Black (NH-Black), Hispanic, other race, Asian, American Indian/Alaska Native, multiracial, and Native Hawaiian/other Pacific Islander. Education level was assessed from the following question, “What is the highest grade or year of school you have completed?” Income was assessed via the question, “Is your annual income from all sources: less than $10,000, or $10,000 to less than $15,000, or $15,000 to less than $20,000, or $20,000 to less than $25,000, or $25,000 to less than $35,000, or $35,000 to less than $50,000, or $50,000 to less than $75,000, or $75,000 or more.” Health insurance status was determined from the question, “Do you have any kind of health care coverage, including health insurance, prepaid plans such as HMOs, or government plans such as Medicare, or Indian Health Service?” We used the following survey question to determine if someone had a primary care doctor, “Do you have one person you think of as your personal doctor or health care provider?” Delayed care due to cost was determined using the survey question, “Was there a time in the last 12 months when you needed to see a doctor but could not because of cost?” We examined geographic data by state divisions defined by the U.S. Census.

Additionally, we examined BRFSS questions on comorbidities including asthma, cardiovascular disease (CVD), hypertension, body mass index (BMI), cancer, and diabetes. Asthma diagnosis was determined by the question, “Has a doctor, nurse, or other health professional ever told you that you had asthma?” CVD diagnosis was determined by the question, “Has a doctor, nurse, or other health professional ever told you that you had angina or coronary heart disease?” Hypertension was determined using the question, “Have you ever been told by a doctor, nurse or other health professional that you have high blood pressure?” BMI was calculated from each participant’s self-reported height and weight. Cancer diagnosis was determined by the question, “Has a doctor, nurse, or other health professional ever told you that you had skin cancer or any other types of cancer?” Diabetes was determined by the survey question, “Has a doctor, nurse, or other health professional ever told you that you had diabetes?”

#### Data analysis

We restricted our analysis to participants who answered the influenza vaccination question and had complete data for covariates (*N* = 279,590). We also limited our analysis to participants in the 50 United States plus the District of Columbia.

We calculated weighted median, interquartile ranges for continuous covariates and weighted percentages for categorical covariates by influenza vaccination status.

To estimate the odds of influenza vaccination we fit fully adjusted logistic regression models for the total population and stratified by race/ethnicity that account for the complex survey design of the data via PROC RLOGIST in SAS-callable (version 9.3, SAS institute, Cary, NC) version of the statistical package SUDAAN (version 11.0.3). Fully adjusted models included the following variables: age, sex, race/ethnicity, education, income, state divisions, health insurance, primary care doctor, delayed care due to cost, asthma, CVD, hypertension, BMI, cancer and diabetes. Tests of statistical significance used two-tailed significance tests at the 0.05 alpha level from Wald F tests calculated in the SUDAAN software to account for the complex survey design.

## Results

The median age of the cohort was 47 years old and included male (51.5%) and female (48.5%) participants. The cohort was NH-White (65.8%), had some college education (32.0%), had an annual income of $75,000 or more (38.7%), were from the South Atlantic division (20.0%), had health insurance (88.7%), had a primary care doctor (78.0%), and had not delayed care due to cost (87.3%). The majority of those surveyed did not have asthma (85.2%), CVD (91.5%), hypertension (67.2%), cancer (87.5%), or diabetes (88.6%). More than one-third of the cohort reported weights and heights in the “overweight” BMI category (35.7%) (Table 1).

**Table 1:** Descriptive Data by Influenza Vaccination, 2019 BRFSS^a^

***N*** (weighted %)^b^				
*N* = 279,590	Total	Percentage who Received Influenza Vaccine	Did not Receive Influenza Vaccine	***P*** value
**Age, median (IQR)**	47 (47, 47)	54 (54, 54)	42 (42, 43)	**< 0.001**
**Sex**				**< 0.001**
Female	144,515 (48.5)	77,208 (46.8)	67,307 (53.3)	
Male	135,075 (51.5)	63,099 (39.7)	71,976 (60.3)	
**Race**				**< 0.001**
NH-White	221,628 (65.8)	116,332 (46.4)	105,296 (53.6)	
NH-Black	20,555 (11.3)	8757 (36.6)	11,798 (63.4)	
Hispanic	18,896 (14.9)	7116 (34.4)	11,780 (65.6)	
Other race	1871 (0.5)	786 (36.7)	1085 (63.3)	
Asian	5516 (5.0)	2594 (44.1)	2922 (55.9)	
American Indian / Alaska Native	4439 (1.0)	1930 (36.5)	2509 (63.5)	
Multiracial	5728 (1.4)	2425 (39.6)	3303 (60.4)	
Native Hawaiian / Other Pacific Islander	957 (0.2)	367 (37.1)	590 (62.9)	
**Education**				**< 0.001**
Less than high school	15,832 (10.4)	6327 (35.0)	9505 (65.0)	
High school graduate	70,399 (26.5)	30,613 (37.0)	39,786 (63.0)	
Some college	79,372 (32.0)	37,675 (41.5)	41,697 (58.6)	
College graduate or more	113,987 (31.1)	65,692 (52.9)	48,295 (47.1)	
**Income**				**< 0.001**
< $25,000	64,505 (23.4)	28,849 (38.7)	35,656 (61.3)	
$25 – $49,999	66,918 (22.4)	32,325 (40.5)	34,593 (59.5)	
$50 – $74,999	46,251 (15.4)	23,425 (42.7)	22,826 (57.3)	
≥ $75,000	101,916 (38.7)	55,708 (47.6)	46,208 (52.5)	
**Divisions**^c^				**< 0.001**
New England	30,529 (4.5)	17,032 (49.9)	13,497 (50.1)	
Middle Atlantic	13,740 (10.0)	7023 (46.5)	6717 (53.5)	
East North Central	30,472 (15.9)	14,916 (41.5)	15,556 (58.5)	
West North Central	51,465 (7.0)	26,979 (46.9)	24,486 (53.1)	
South Atlantic	49,743 (20.0)	25,157 (43.1)	24,586 (56.9)	
East South Central	17,111 (5.8)	8091 (41.2)	9020 (58.9)	
West South Central	19,033 (12.0)	9384 (41.0)	9649 (59.0)	
Mountain	38,722 (7.7)	18,258 (41.4)	20,464 (58.6)	
Pacific	28,775 (17.1)	13,467 (42.4)	15,308 (57.6)	
**Health Insurance**				**< 0.001**
Yes	258,317 (88.7)	135,989 (46.3)	122,328 (53.7)	
No	21,273 (11.3)	4318 (18.6)	16,955 (81.4)	
**Primary Care Doctor**				**< 0.001**
Yes	234,026 (78.0)	128,929 (48.9)	105,097 (51.1)	
No	45,564 (22.0)	11,378 (22.6)	34,186 (77.4)	
**Delayed Care due to Cost**				**< 0.001**
No	251,177 (87.3)	130,907 (45.3)	120,270 (54,7)	
Yes	28,413 (12.7)	9400 (28.5)	19,013 (71.5)	
**Asthma**				**< 0.001**
No	240,107 (85.2)	119,322 (42.6)	120,785 (57.5)	
Yes	39,483 (14.8)	20,985 (46.6)	18,498 (53.4)	
**CVD**				**< 0.001**
No	247,031 (91.5)	120,489 (42.0)	126,542 (58.1)	
Yes	32,559 (8.5)	19,818 (56.0)	12,741 (44.0)	
**Hypertension**				**< 0.001**
No	166,058 (67.2)	74,164 (38.6)	91,894 (61.4)	
Yes	113,532 (32.8)	66,143 (52.5)	47,389 (47.5)	
**BMI**				**< 0.001**
Normal/underweight	87,245 (32.5)	42,587 (41.9)	44,658 (58.1)	
Overweight	100,609 (35.7)	50,953 (43.3)	49,656 (56.7)	
Obese	91,736 (31.8)	46,767 (44.3)	44,969 (55.7)	
**Cancer**				**< 0.001**
No	228,301 (87.5)	107,429 (40.7)	120,872 (59.3)	
Yes	51,289 (12.5)	32,878 (60.2)	18,411 (39.8)	
**Diabetes**				**< 0.001**
No	240,133 (88.6)	115,718 (41.3)	124,415 (58.8)	
Yes	39,457 (11.4)	24,589 (57.8)	14,868 (42.2)	

Notes: Boldface indicates statistical significance *p* < 0.05

^a^ Includes data from the 2019 BRFSS annual survey. Analysis excludes US territories

^b^ Data represents N (weighted percentage) unless otherwise noted

^c^ West North Central division includes: Iowa, Kansas, Minnesota, Missouri, Nebraska, North Dakota, and South Dakota. New England division includes: Connecticut, Maine, Massachusetts, New Hampshire, Rhode Island, and Vermont. Middle Atlantic includes: New Jersey, New York, and Pennsylvania. South Atlantic division includes: Delaware, District of Columbia, Florida, Georgia, Maryland, North Carolina, South Carolina, Virginia, and West Virginia. Mountain division includes: Arizona, Colorado, Idaho, New Mexico, Montana, Utah, Nevada, and Wyoming. Pacific division includes: Alaska, California, Hawaii, Oregon, and Washington. East North Central division includes: Indiana, Illinois, Michigan, Ohio, and Wisconsin. East South Central division includes: Alabama, Kentucky, Mississippi, and Tennessee. West South Central division includes: Arkansas, Louisiana, Oklahoma, and Texas

Participants who received the influenza vaccine were more likely to be older (median age 54 years old, compared to 42 years old for those not vaccinated), and female (46.8% of females vaccinated compared to 39.7% of males vaccinated). Asian (44.1%) and NH-White (46.4%) race/ethnicity groups had a higher percentage of their population receive the influenza vaccine compared to NH-Black (36.6%), Hispanic (34.4%), American Indian/Alaska native (36.5%), and Native Hawaiian / Other Pacific Islander groups (37.1%). Influenza vaccination uptake was higher in individuals with at least a college degree (52.9%) compared to those with less than a high school education (35.0%) and higher in individuals with an annual income of $75,000 or more (47.6%) compared to those with an annual income less than $25,000 (38.7%). Participants in New England had the highest vaccine coverage (49.9%) compared to the West South-Central division which had the lowest (41.0%).

Influenza vaccine uptake was more common among those with comorbidities. Participants with asthma (46.6%), CVD (56.0%), hypertension (52.5%), cancer (60.2%) and diabetes (57.8%) had higher vaccine uptake than those without (42.6, 42.0, 38.6, 40.7, and 41.3% respectively). Those in the overweight (43.3%) and obese (44.3%) BMI categories were more likely to receive the influenza vaccine compared to those in the underweight (41.9%) category (Table 1).

Supplemental tables show similar descriptive associations of covariates by influenza vaccination within race/ethnicity groups.

**Fig. 1:**
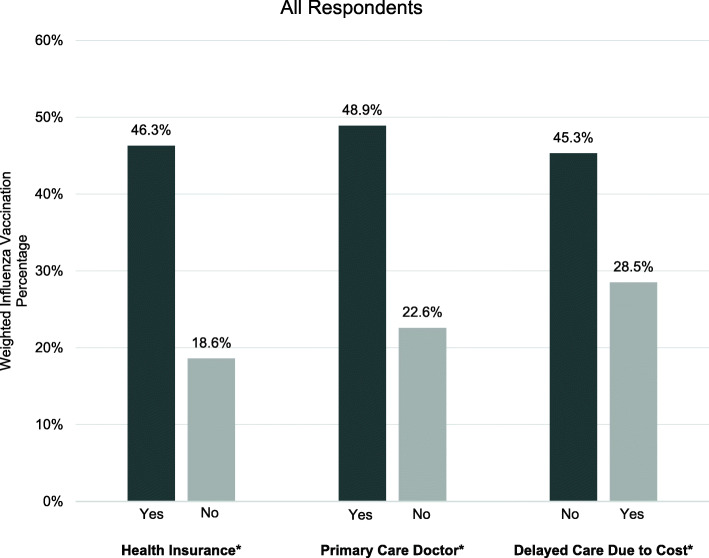
Impact of Structural Factors on Influenza Vaccination. Notes: (*p < 0.001)

**Fig. 2:**
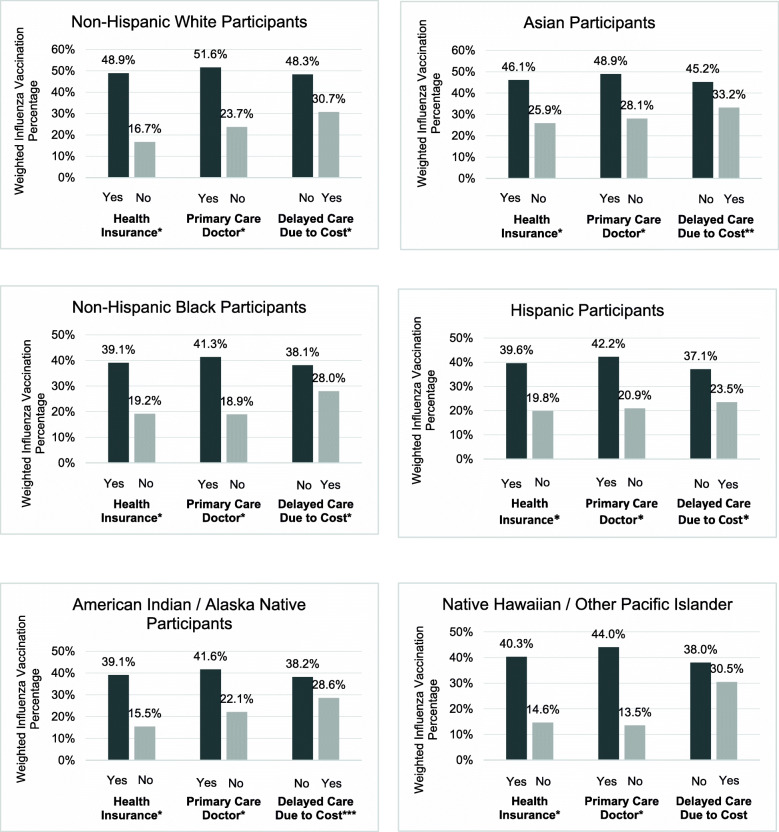
Impact of Structural Factors on Influenza Vaccination by Race/Ethnicity. Notes: (*p < 0.001, **p = 0.004, ***p = 0.011)

Figure 1 shows the weighted prevalence of influenza vaccination by structural factors. Individuals who have health insurance (46.3%), have a primary care doctor (48.9%), or do not have delayed care due to cost (45.3%) had higher uptake of influenza vaccination compared to those who did not (18.6, 22.6 and 28.5%, respectively). In Fig. 2 we see similar trends in the weighted prevalence of influenza vaccination by structural factors, within groups stratified by race/ethnicity. For NH-White, NH-Black, Hispanic, Asian, and American Indian/Alaska Native individuals, those who have health insurance, have a primary care doctor, or do not have delayed care due to cost had higher uptake of influenza vaccination compared to those who did not. For Native Hawaiian/other Pacific Islanders, those who have health insurance or have a primary care doctor had a higher uptake of influenza vaccination compared to those who did not.

In weighted logistic regression models predicting influenza vaccination (Table 2), after adjusting for covariates, older participants were more likely to receive the influenza vaccine [OR: 1.01 (1.01, 1.02)]. Males [OR: 0.81 (0.78, 0.83)] were less likely to receive the influenza vaccine than females. NH-Black individuals [OR: 0.75 (0.71, 0.79)], and American Indian/Alaska Natives [OR: 0.76 (0.66, 0.88)] were less likely to receive the influenza vaccine compared to NH-White individuals. Individuals without health insurance [OR: 0.52 (0.48, 0.56)], without a primary care doctor [OR: 0.52 (0.50, 0.55)] or who had delayed medical care due to cost [OR: 0.71 (0.67, 0.75)] were less likely to receive the influenza vaccine. Individuals with less than high school education [OR: 0.57 (0.53, 0.62)] were less likely to receive the influenza vaccine compared to those with a college degree or more. The lowest income group, less than $25,000 [OR: 0.94 (0.90, 0.99)], was less likely to receive the influenza vaccine than the highest income group, $75,000 or more. All the divisions except for the Middle Atlantic and West North Central were less likely to receive the influenza vaccine compared to individuals in the New England division. Individuals with asthma [OR: 1.20 (1.15, 1.26)], CVD [OR: 1.13 (1.07, 1.19)], hypertension [OR: 1.25 (1.21, 1.30)], cancer [OR: 1.34 (1.28, 1.40)], or diabetes [OR: 1.40 (1.33, 1.47)] were all more likely to receive the influenza vaccine compared to those without these comorbidities.

**Table 2:** Odds Ratios from Weighted Logistic Regression Predicting Influenza Vaccination, 2019 BRFSS^a^

Odds Ratio (95% CI)^b^		
*N* = 279,590	Odds Ratio (95% CI)	***P*** value
**Age**	1.01 (1.01, 1.02)	**< 0.001**
**Sex**		**< 0.001**
Female	1.00	
Male	0.81 (0.78, 0.83)	
**Race**		**< 0.001**
NH-White	1.00	
NH-Black	0.75 (0.71, 0.79)	
Hispanic	1.02 (0.95, 1.09)	
Other race	0.75 (0.60, 0.93)	
Asian	1.06 (0.94, 1.19)	
American Indian / Alaska Native	0.76 (0.66, 0.88)	
Multiracial	0.92 (0.82, 1.02)	
Native Hawaiian / Other Pacific Islander	0.87 (0.65, 1.17)	
**Education**		**< 0.001**
Less than high school	0.57 (0.53, 0.62)	
High school graduate	0.58 (0.55, 0.60)	
Some college	0.67 (0.64, 0.70)	
College graduate or more	1.00	
**Income**		**0.005**
< $25,000	0.94 (0.90, 0.99)	
$25 – $49,999	0.94 (0.90, 0.99)	
$50 – $74,999	0.93 (0.88, 0.97)	
≥ $75,000	1.00	
**Divisions**^**c**^		**< 0.001**
New England	1.00	
Middle Atlantic	0.95 (0.89, 1.01)	
East North Central	0.79 (0.75, 0.83)	
West North Central	1.02 (0.97, 1.08)	
South Atlantic	0.88 (0.83, 0.93)	
East South Central	0.84 (0.79, 0.90)	
West South Central	0.90 (0.84, 0.98)	
Mountain	0.84 (0.79, 0.89)	
Pacific	0.86 (0.80, 0.91)	
**Health Insurance**		**< 0.001**
Yes	1.00	
No	0.52 (0.48, 0.56)	
**Primary Care Doctor**		**< 0.001**
Yes	1.00	
No	0.52 (0.50, 0.55)	
**Delayed Care due to Cost**		
No	1.00	**< 0.001**
Yes	0.71 (0.67, 0.75)	
**Asthma**		
No	1.00	**< 0.001**
Yes	1.20 (1.15, 1.26)	
**CVD**		
No	1.00	**< 0.001**
Yes	1.13 (1.07, 1.19)	
**Hypertension**		
No	1.00	**< 0.001**
Yes	1.25 (1.21, 1.30)	
**BMI**		0.285
Normal/under	1.00	
Overweight	0.97 (0.93, 1.01)	
Obese	0.98 (0.94, 1.02)	
**Cancer**		**< 0.001**
No	1.00	
Yes	1.34 (1.28, 1.40)	
**Diabetes**		**< 0.001**
No	1.00	
Yes	1.40 (1.33, 1.47)	

Notes: Boldface indicates statistical significance *p* < 0.05

^a^ Includes data from the 2019 BRFSS annual survey. Analysis excludes US territories

^b^ Data represent odds ratios (95% confidence intervals)

^c^ New England division includes: Connecticut, Maine, Massachusetts, New Hampshire, Rhode Island, and Vermont. Middle Atlantic includes: New Jersey, New York, and Pennsylvania. East North Central division includes: Indiana, Illinois, Michigan, Ohio, and Wisconsin. West North Central division includes: Iowa, Kansas, Minnesota, Missouri, Nebraska, North Dakota, and South Dakota. South Atlantic division includes: Delaware, District of Columbia, Florida, Georgia, Maryland, North Carolina, South Carolina, Virginia, and West Virginia. East South Central division includes: Alabama, Kentucky, Mississippi, and Tennessee. West South Central division includes: Arkansas, Louisiana, Oklahoma, and Texas. Mountain division includes: Arizona, Colorado, Idaho, New Mexico, Montana, Utah, Nevada, and Wyoming. Pacific division includes: Alaska, California, Hawaii, Oregon, and Washington

When stratifying models for NH-White individuals, males [OR: 0.79 (0.77, 0.82)], those with less than a high school education [OR: 0.42 (0.38, 0.46)], with less than $25,000 annual income [OR: 0.89 (0.84, 0.93)], without a primary care doctor [OR: 0.49 (0.47, 0.52)], without health insurance [OR: 0.41 (0.38, 0.44)], or had delayed medical care due to cost [OR: 0.73 (0.68, 0.77)] were less likely to receive the influenza vaccine. NH-White individuals with asthma [1.20 (1.14, 1.26)], CVD [1.13 (1.07, 1.20)], hypertension [1.27 (1.23, 1.32)], cancer [1.33 (1.27, 1.39)], or diabetes [1.42 (1.34, 1.50)] had higher vaccine uptake than those without (Table 3). For NH-Black individuals, males [OR: 0.88 (0.79, 0.98)], those with less than a high school education [OR: 0.75 (0.60, 0.93)], without a primary care doctor [OR: 0.53 (0.45, 0.63)], and without health insurance [OR: 0.61 (0.49, 0.76)] were less likely to receive the influenza vaccine. NH-Black individuals with asthma [1.29 (1.12, 1.50)], hypertension [1.28 (1.14, 1.44)], cancer [1.49 (1.24, 1.80)], or diabetes [1.51 (1.32, 1.73)] had higher vaccine uptake than those without. For Hispanic individuals, males [OR: 0.80 (0.71, 0.91)], those with less than a high school education [OR: 0.79 (0.65, 0.96)], without a primary care doctor [OR: 0.54 (0.47, 0.63)], without health insurance [OR: 0.59 (0.50, 0.69)], or had delayed medical care due to cost [OR: 0.65 (0.55, 0.76)] were less likely to receive the influenza vaccine. Hispanic individuals with asthma [OR: 1.23 (1.04, 1.45)], cancer [OR: 1.28 (1.00, 1.64)], or diabetes [OR: 1.30 (1.09, 1.56)] had higher vaccine uptake than those without. For Asian individuals, those without a primary care doctor [OR: 0.56 (0.42, 0.74)], and without health insurance [OR: 0.63 (0.40, 0.99)] were less likely to receive the influenza vaccine. For American Indian/Alaska Natives models, males [OR: 0.72 (0.55, 0.93)], those without a primary care doctor [OR: 0.60 (0.45, 0.81)], and without health insurance [OR: 0.45 (0.28, 0.71)] were less likely to receive the influenza vaccine. American Indian/Alaska Natives individuals with asthma [OR: 1.56 (1.08, 2.25)] had higher vaccine uptake than those without. For Native Hawaiian/other Pacific Islander individuals, those without a primary care doctor [OR: 0.23 (0.12, 0.44)] were less likely to receive the influenza vaccine than those without.

**Table 3:** Odds Ratios from Weighted Logistic Regression Predicting Influenza Vaccination by Race/Ethnicity, 2019 BRFSS^a^

	NH White ***N*** = 221,628	NH Black ***N*** = 20,555		Hispanic ***N*** = 18,896		Asian ***N*** = 5516		American Indian and Alaska Native ***N*** = 4439		Native Hawaiian or Other Pacific Islander ***N*** = 957	
	***P*** **value**	***P*** **value**		***P*** **value**		***P*** **value**		***P*** **value**		***P*** **value**	
	**OR (95% CI)**	**OR (95% CI)**		**OR (95% CI)**		**OR (95% CI)**		**OR (95% CI)**		**OR (95% CI)**	
**Age**	**< 0.001**	**< 0.001**		**< 0.001**		**0.010**		**0.002**		0.783	
	1.02 (1.01, 1.02)	1.01 (1.01, 1.02)		1.01 (1.01, 1.02)		1.01 (1.00, 1.02)		1.02 (1.01, 1.02)		1.00 (0.98, 1.02)	
**Sex**	**< 0.001**	**0.016**		**< 0.001**		0.215		**0.011**		0.153	
Female	1.00	1.00		1.00		1.00		1.00		1.00	
Male	0.79 (0.77, 0.82)	0.88 (0.79, 0.98)		0.80 (0.71, 0.91)		0.87 (0.70, 1.08)		0.72 (0.55, 0.93)		0.68 (0.40, 1.15)	
**Education**	**< 0.001**	**< 0.001**		**< 0.001**		0.096		**0.024**		0.476	
Less than high school	0.42 (0.38, 0.46)	0.75 (0.60, 0.93)		0.79 (0.65, 0.96)		1.46 (0.62, 3.43)		0.75 (0.46, 1.20)		0.46 (0.16, 1.34)	
High school graduate	0.54 (0.51, 0.56)	0.71 (0.61, 0.83)		0.68 (0.57, 0.81)		0.74 (0.54, 1.02)		0.59 (0.40, 0.88)		0.90 (0.42, 1.92)	
Some college	0.64 (0.61, 0.66)	0.80 (0.69, 0.91)		0.77 (0.65, 0.92)		0.78 (0.57, 1.05)		0.59 (0.41, 0.85)		0.74 (0.37, 1.51)	
College graduate or more	1.00	1.00		1.00		1.00		1.00		1.00	
**Income**	**< 0.001**	0.156		0.331		0.259		0.933		0.724	
< $25,000	0.89 (0.84, 0.93)	1.01 (0.86, 1.20)		1.18 (0.99, 1.40)		0.86 (0.62, 1.20)		1.12 (0.77, 1.62)		1.22 (0.62, 2.39)	
$25 – $49,999	0.93 (0.88, 0.97)	1.09 (0.93, 1.28)		1.08 (0.91, 1.29)		0.72 (0.52, 0.99)		1.10 (0.72, 1.68)		1.30 (0.67, 2.53)	
$50 – $74,999	0.92 (0.88, 0.97)	0.89 (0.74, 1.06)		1.07 (0.86, 1.32)		0.88 (0.63, 1.21)		1.14 (0.70, 1.84)		1.64 (0.66, 4.09)	
≥ $75,000	1.00	1.00		1.00		1.00		1.00		1.00	
**Divisions**^**b**^	**< 0.001**	**< 0.001**		**< 0.001**		0.949		**0.003**		0.173	
New England	1.00	1.00		1.00		1.00		1.00		1.00	
Middle Atlantic	0.96 (0.90, 1.03)	0.85 (0.66, 1.10)		0.95 (0.76, 1.20)		0.86 (0.57, 1.31)		1.28 (0.54, 3.04)		0.380.09, 1.53)	
East North Central	0.81 (0.76, 0.85)	0.57 (0.45, 0.73)		0.75 (0.60, 0.93)		1.14 (0.76, 1.71)		0.92 (0.42, 2.03)		0.45 (0.09, 2.40)	
West North Central	1.03 (0.98, 1.09)	0.90 (0.69, 1.17)		0.97 (0.77, 1.21)		1.08 (0.72, 1.60)		1.69 (0.85, 3.34)		1.92 (0.48, 7.73)	
South Atlantic	0.93 (0.88, 0.99)	0.75 (0.60, 0.93)		0.68 (0.53, 0.87)		1.02 (0.68, 1.53)		1.14 (0.57, 2.27)		0.89 (0.25, 3.09)	
East South Central	0.86 (0.80, 0.92)	0.72 (0.57, 0.91)		1.32 (0.87, 1.99)		0.84 (0.36, 1.94)		1.01 (0.48, 2.13)		0.42 (0.11, 1.17)	
West South Central	0.87 (0.80, 0.95)	0.79 (0.60, 1.04)		1.00 (0.78, 1.28)		0.95 (0.57, 1.58)		1.43 (0.68, 2.98)		0.89 (0.17, 4.53)	
Mountain	0.84 (0.79, 0.89)	0.72 (0.53, 1.00)		0.81 (0.66, 0.98)		0.92 (0.58, 1.46)		2.14 (1.10, 4.19)		0.31 (0.09, 1.03)	
Pacific	0.84 (0.78, 0.90)	0.67 (0.49, 0.91)		0.82 (0.68, 1.00)		1.04 (0.73, 1.46)		1.40 (0.68, 2.88)		0.62 (0.24, 1.63)	
**Health Insurance**	**< 0.001**	**< 0.001**		**< 0.001**		**0.049**		**0.001**		0.152	
Yes	1.00	1.00		1.00		1.00		1.00		1.00	
No	0.41 (0.38, 0.44)	0.61 (0.49, 0.76)		0.59 (0.50, 0.69)		0.63 (0.40, 0.99)		0.45 (0.28, 0.71)		0.54 (0.23, 1.26)	
**Primary Care Doctor**	**< 0.001**	**< 0.001**		**< 0.001**		**< 0.001**		**0.001**		**< 0.001**	
Yes	1.00	1.00		1.00		1.00		1.00		1.00	
No	0.49 (0.47, 0.52)	0.53 (0.45, 0.63)		0.54 (0.47, 0.63)		0.56 (0.42, 0.74)		0.60 (0.45, 0.81)		0.23 (0.12, 0.44)	
**Delayed Care due to Cost**	**< 0.001**	**0.003**		**< 0.001**		0.145		**0.040**		0.971	
No	1.00	1.00		1.00		1.00		1.00		1.00	
Yes	0.73 (0.68, 0.77)	0.79 (0.68, 0.92)		0.65 (0.55, 0.76)		0.76 (0.52, 1.10)		0.70 (0.50, 0.98)		0.98 (0.41, 2.36)	
**Asthma**	**< 0.001**	**< 0.001**		**0.018**		0.457		**0.017**		0.901	
No	1.00	1.00		1.00		1.00		1.00		1.00	
Yes	1.20 (1.14, 1.26)	1.29 (1.12, 1.50)		1.23 (1.04, 1.45)		1.14 (0.80, 1.62)		1.56 (1.08, 2.25)		1.04 (0.57, 1.91)	
**CVD**	**< 0.001**	0.377		0.158		0.127		0.078		0.202	
No	1.00	1.00		1.00		1.00		1.00		1.00	
Yes	1.13 (1.07, 1.20)	1.08 (0.91, 1.27)		1.19 (0.94, 1.51)		1.55 (0.88, 2.72)		1.41 (0.96, 2.07)		1.61 (0.78, 3.34)	
**Hypertension**	**< 0.001**	**< 0.001**		0.060		0.070		0.195		0.165	
No	1.00	1.00		1.00		1.00		1.00		1.00	
Yes	1.27 (1.23, 1.32)	1.28 (1.14, 1.44)		1.15 (0.99, 1.34)		1.31 (0.98, 1.77)		1.22 (0.90, 1.65)		1.48 (0.85, 2.58)	
**BMI**	0.235	0.910		0.627		0.412		0.174		0.469	
Normal/under	1.00	1.00		1.00		1.00		1.00		1.00	
Overweight	0.96 (0.93, 1.01)	0.98 (0.85, 1.14)		1.07 (0.92, 1.24)		0.86 (0.68, 1.09)		0.70 (0.49, 1.02)		0.72 (0.34, 1.49)	
Obese	0.98 (0.94, 1.02)	0.98 (0.84, 1.12)		1.02 (0.88, 1.18)		1.02 (0.72, 1.45)		0.86 (0.62, 1.18)		0.99 (0.48, 2.05)	
**Cancer**	**< 0.001**	**< 0.001**		**0.046**		0.568		0.751		0.423	
No	1.00	1.00		1.00		1.00		1.00		1.00	
Yes	1.33 (1.27, 1.39)	1.49 (1.24, 1.80)		1.28 (1.00, 1.64)		1.25 (0.58, 2.67)		0.93 (0.59, 1.47)		1.49 (0.56, 3.98)	
**Diabetes**	**< 0.001**	**< 0.001**		**0.004**		0.444		0.131		0.663	
No	1.00	1.00		1.00		1.00		1.00		1.00	
Yes	1.42 (1.34, 1.50)	1.51 (1.32, 1.73)		1.30 (1.09, 1.56)		1.17 (0.78, 1.76)		1.33 (0.92, 1.93)		0.86 (0.44, 1.68)	

Notes: Boldface indicates statistical significance *p* < 0.05

^a^ Includes data from the 2019 BRFSS annual survey. Analysis excludes US territories

^b^ New England division includes: Connecticut, Maine, Massachusetts, New Hampshire, Rhode Island, and Vermont. Middle Atlantic includes: New Jersey, New York, and Pennsylvania. East North Central division includes: Indiana, Illinois, Michigan, Ohio, and Wisconsin. West North Central division includes: Iowa, Kansas, Minnesota, Missouri, Nebraska, North Dakota, and South Dakota. South Atlantic division includes: Delaware, District of Columbia, Florida, Georgia, Maryland, North Carolina, South Carolina, Virginia, and West Virginia. East South Central division includes: Alabama, Kentucky, Mississippi, and Tennessee. West South Central division includes: Arkansas, Louisiana, Oklahoma, and Texas. Mountain division includes: Arizona, Colorado, Idaho, New Mexico, Montana, Utah, Nevada, and Wyoming. Pacific division includes: Alaska, California, Hawaii, Oregon, and Washington

## Discussion

This analysis illustrates the many structural and associated demographic factors that contribute to seasonal influenza vaccination uptake. The racial and ethnic disparities in influenza vaccine uptake are of particular concern amidst the larger context of persistent health inequities among marginalized groups [14].

Structural factors matter when it comes to influenza vaccination rates. Those with less access to preventive health care, as measured by the lack of a primary care doctor, the lack of health insurance, and a history of delaying care due to cost, are also less likely to receive the flu vaccine. The increased likelihood of vaccine uptake among those with comorbidities is likely due to increased contact with the healthcare system for treatment of disease [5, 15]. These healthcare access factors are further connected to income status, as those in lower income brackets are likewise less likely to receive the flu vaccine. Consistent with other literature, we find that health care access alone does not fully explain vaccination inequities in some groups, particularly among African American/Black survey respondents. A recent study of influenza vaccine uptake among the US Medicare population found that while vaccine uptake was below recommended levels across the cohort, the racial disparity was persistent [16]. The “Unequal Treatment” model defined by the National Academy of Medicine acknowledges that access to healthcare is indicative of larger structural forces within health care, including provider bias [10]. Importantly, additional structural factors outside of health care, including residential segregation, wealth, employment-related factors, and other conditions may also contribute [11]. An examination of these important, broader social and structural inequities are not addressed with these data.

A limitation of these data is that the BRFSS does not have information on attitudes towards influenza vaccination, including concern about side effects [6], low perception of risk regarding influenza infection, perceived ineffectiveness of the vaccine, [5] and distrust of physicians [6], which have been described as contributors to vaccine hesitancy and the persistence of racial and ethnic care disparities. However, it is critical to note that issues of influenza vaccine hesitancy are also related to structural inequities in access to care. Strategies that have demonstrated success in responding to the complex issue of vaccine hesitancy and increased influenza vaccine uptake indicate the importance of multi-component interventions [17]. When patients have access to a primary care clinician, an emphasis on conversations with healthcare clinicians about the health benefit and importance of influenza vaccination, with acknowledgments of concerns and side effects helps to increase uptake while also fostering trust between patients and healthcare providers, which is of particular benefit to African American and Latina/o populations [18]. Linguistically-relevant, dialogue-based interventions used along with lowering out-of-pocket cost, standing orders, reminder systems for provider and client, community vaccine programs and other community-based interventions all have proven to increase vaccine uptake [19].

While this study is strengthened by the size of the cohort, which surveys a nationally representative sample of the U.S. adult population, a limitation is its generalizability beyond the adult population and racial/ethnic groups sampled through the BRFSS. As an additional limitation, understanding racial and ethnic differences in vaccination can be complicated by important heterogeneity within racial or ethnic subgroups—for example, in the heterogenous subgroups classified as Asian—which can mask differences within subgroups and lead to unaddressed structural inequities in these groups. Moreover, the BRFSS is a telephone survey that relies on self-reported data to measure vaccination uptake [20]. A strength of the BRFSS is the use of validated questions on influenza vaccine receipt [21]. Additional studies are needed to capture data on populations that are not reached by landline telephone surveys.

## Conclusion

There are multiple persistent structural inequities related to accessing care and paying for care that influence influenza vaccination rates for Black, Indigenous, people of color, low-income populations and other groups in the United States. Several strategies to mitigate these structural factors have been successful in past efforts to increase vaccination rates, though more fundamental efforts to increase access to care and address financial barriers to care are needed. It is essential that policy efforts to increase vaccination rates address access to care as fundamental structural factors that impede vaccination, in order to mitigate influenza uptake disparities, and to stem inequities seen in newer campaigns as the U.S. distributes the COVID-19 vaccine.

## Supplementary Information

**Additional file 1: Supplemental Table 1.** Non-Hispanic White Descriptive Data by Influenza Vaccination, 2019 BRFSS. **Supplemental Table 2.** Non-Hispanic Black Descriptive Data by Influenza Vaccination, 2019 BRFSS. **Supplemental Table 3.** Hispanic Descriptive Data by Influenza Vaccination, 2019 BRFSS. **Supplemental Table 4.** Asian Descriptive Data by Influenza Vaccination, 2019 BRFSS. **Supplemental Table 5.** American Indian / Alaska Native Descriptive Data by Influenza Vaccination, 2019 BRFSS. **Supplemental Table 6.** Native Hawaiian or Other Pacific Islander Descriptive Data by Influenza Vaccination, 2019 BRFSS.

## Data Availability

The dataset used for this analysis is available for public access from the Behavioral Risk Factor Surveillance System: https://www.cdc.gov/brfss/annual_data/annual_2019.html

## Abbreviations

CDC: Centers for Disease Control and Prevention
BRFSS: Behavioral Risk Factor Surveillance System
COVID-19: Coronavirus disease 2019
