# Supplementary material for: Structural inequities in seasonal influenza vaccination rates
Source: BMC Public Health. 2021 Jun 17;21:1166. doi: 10.1186/s12889-021-11179-9 (PMC8210739; doi:10.1186/s12889-021-11179-9)
Supplement: Supplementary file 1 — Additional file 1: Supplemental Table 1. Non-Hispanic White Descriptive Data by Influenza Vaccination, 2019 BRFSS. Supplemental Table 2. Non-Hispanic Black Descriptive Data by Influenza Vaccination, 2019 BRFSS. Supplemental Table 3. Hispanic Descriptive Data by Influenza Vaccination, 2019 BRFSS. Supplemental Table 4. Asian Descriptive Data by Influenza Vaccination, 2019 BRFSS. Supplemental Table 5. American Indian / Alaska Native Descriptive Data by Influenza Vaccination, 2019 BRFSS. Supplemental Table 6. Native Hawaiian or Other Pacific Islander Descriptive Data by Influenza Vaccination, 2019 BRFSS. [file 12889_2021_11179_MOESM1_ESM.docx]

| **Supplemental Table 1. Non-Hispanic White Descriptive Data by Influenza Vaccination, 2019 BRFSS** | | | | |
| --- | --- | --- | --- | --- |
| **N (weighted %)** | | | | |
| N= 221628 | **Total** | **Percentage who Received Influenza Vaccine** | **Did not Receive Influenza Vaccine** | **P value** |
| **Age, median (IQR)** | 51 (50, 51) | 57 (57, 57) | 45 (45, 46) | **<0.001** |
| **Sex** |  |  |  | **<0.001** |
| Female | 113987 (48.4) | 63567 (50.4) | 50420 (49.6) |  |
| Male | 107641 (51.6) | 52765 (42.7) | 54876 (57.3) |  |
| **Education** |  |  |  | **<0.001** |
| Less than high school | 8605 (6.5) | 3532 (33.8) | 5073 (66.2) |  |
| High school graduate | 54442 (26.4) | 24745 (39.7) | 29697 (60.4) |  |
| Some college | 63372 (33.1) | 31303 (44.3) | 32069 (55.7) |  |
| College graduate or more | 95209 (33.9) | 56752 (56.2) | 38457 (43.8) |  |
| **Income** |  |  |  | **<0.001** |
| < $25,000 | 43583 (17.9) | 20606 (41.8) | 22977 (58.2) |  |
| $25 – $49,999 | 52266 (21.4) | 26608 (44.2) | 25658 (55.8) |  |
| $50 – $74,999 | 38449 (16.7) | 20193 (45.4) | 18256 (54.6) |  |
| ≥ $75,000 | 87330 (44.0) | 48925 (49.8) | 38405 (50.2) |  |
| **Divisions** |  |  |  | **<0.001** |
| New England | 27246 (5.5) | 15557 (51.7) | 11689 (48.3) |  |
| Middle Atlantic | 10522 (10.4) | 5628 (49.3) | 4894 (50.7) |  |
| East North Central | 25747 (18.6) | 13136 (43.9) | 12611 (56.2) |  |
| West North Central | 45681 (9.0) | 24539 (48.6) | 21142 (51.4) |  |
| South Atlantic | 36438 (19.3) | 19592 (47.5) | 16846 (52.5) |  |
| East South Central | 13022 (6.7) | 6427 (43.3) | 6595 (56.6) |  |
| West South Central | 13197 (9.8) | 6945 (44.8) | 6252 (55.2) |  |
| Mountain | 31224 (8.1) | 15212 (44.1) | 16012 (55.8) |  |
| Pacific | 18551 (12.7) | 9296 (46.7) | 9255 (53.3) |  |
| **Health Insurance** |  |  |  | **<0.001** |
| Yes | 208625 (92.4) | 113753 (48.9) | 94872 (51.1) |  |
| No | 13003 (7.7) | 2579 (16.7) | 10424 (83.3) |  |
| **Primary Care Doctor** |  |  |  | **<0.001** |
| Yes | 189685 (81.4) | 108114 (51.6) | 81572 (48.4) |  |
| No | 31943 (18.6) | 8218 (23.7) | 23725 (76.3) |  |
| **Delayed Care due to Cost** |  |  |  | **<0.001** |
| No | 202121 (89.3) | 109603 (48.3) | 92518 (51.7) |  |
| Yes | 19507 (10.7) | 6729 (30.7) | 12778 (69.3) |  |
| **Asthma** |  |  |  | **<0.001** |
| No | 191329 (85.3) | 99553 (46.0) | 91776 (54.1) |  |
| Yes | 30299 (14.7) | 16779 (49.2) | 13520 (50.8) |  |
| **CVD** |  |  |  | **<0.001** |
| No | 195004 (90.8) | 99681 (45.2) | 95323 (54.8) |  |
| Yes | 26624 (9.2) | 16651 (58.4) | 9973 (41.6) |  |
| **Hypertension** |  |  |  | **<0.001** |
| No | 130930 (65.9) | 61605 (41.8) | 69325 (58.2) |  |
| Yes | 90698 (34.1) | 54727 (55.4) | 35971 (44.6) |  |
| **BMI** |  |  |  | **<0.001** |
| Normal/underweight | 70600 (33.3) | 36018 (44.7) | 34582 (55.3) |  |
| Overweight | 80621 (35.9) | 42708 (46.6) | 37913 (53.4) |  |
| Obese | 70407 (30.8) | 37606 (48.0) | 32801 (52.0) |  |
| **Cancer** |  |  |  | **<0.001** |
| No | 175151 (83.9) | 86168 (43.5) | 89023 (56.5) |  |
| Yes | 46477 (16.1) | 30204 (61.6) | 16273 (38.4) |  |
| **Diabetes** |  |  |  | **<0.001** |
| No | 192428 (89.4) | 97473 (44.7) | 94955 (55.35) |  |
| Yes | 29200 (10.6) | 18859 (61.5) | 10341 (38.5) |  |

Notes: Boldface indicates statistical significance *p*<0.05.

Notes: Data represent N (weighted percentage) unless otherwise noted Includes data from the 2019 BRFSS annual survey. Analysis includes Non-Hispanic White participants who completed the 2019 survey from January 2019-April 2020. Analysis excludes US territories. West North Central division includes: Iowa, Kansas, Minnesota, Missouri, Nebraska, North Dakota, and South Dakota. New England division includes: Connecticut, Maine, Massachusetts, New Hampshire, Rhode Island, and Vermont. Middle Atlantic includes: New Jersey, New York, and Pennsylvania. South Atlantic division includes: Delaware, District of Columbia, Florida, Georgia, Maryland, North Carolina, South Carolina, Virginia, and West Virginia. Mountain division includes: Arizona, Colorado, Idaho, New Mexico, Montana, Utah, Nevada, and Wyoming. Pacific division includes: Alaska, California, Hawaii, Oregon, and Washington. East North Central division includes: Indiana, Illinois, Michigan, Ohio, and Wisconsin. East South Central division includes: Alabama, Kentucky, Mississippi, and Tennessee. West South Central division includes: Arkansas, Louisiana, Oklahoma, and Texas.

| **Supplemental Table 2. Non-Hispanic Black Descriptive Data by Influenza Vaccination, 2019 BRFSS** | | | | |
| --- | --- | --- | --- | --- |
| **N (weighted %)** | | | | |
| N= 20555 | **Total** | **Percentage who Received Influenza Vaccine** | **Did not Receive Influenza Vaccine** | **P value** |
| **Age, median (IQR)** | 45 (45, 46) | 52 (51, 53) | 42 (41, 43) | **<0.001** |
| **Sex** |  |  |  | **<0.001** |
| Female | 12165 (52.3) | 5417 (38.8) | 6748 (61.2) |  |
| Male | 8390 (47.7) | 3340 (34.1) | 5050 (65.9) |  |
| **Education** |  |  |  | **<0.001** |
| Less than high school | 1923 (11.0) | 864 (39.2) | 1059 (60.7) |  |
| High school graduate | 6139 (30.3) | 2437 (33.1) | 3702 (66.9) |  |
| Some college | 5879 (34.0) | 2379 (35.0) | 3500 (65.0) |  |
| College graduate or more | 6614 (24.8) | 3077 (41.7) | 3537 (58.3) |  |
| **Income** |  |  |  | 0.143 |
| < $25,000 | 8022 (35.4) | 3328 (35.9) | 4694 (64.1) |  |
| $25 – $49,999 | 5153 (24.4) | 2175 (36.7) | 2978 (63.3) |  |
| $50 – $74,999 | 2768 (14.1) | 1164 (34.2) | 1604 (65.8) |  |
| ≥ $75,000 | 4612 (26.1) | 2090 (38.6) | 2522 (61.4) |  |
| **Divisions** |  |  |  | **<0.001** |
| New England | 847 (2.2) | 400 (44.4) | 447 (55.6) |  |
| Middle Atlantic | 1284 (10.6) | 587 (41.1) | 697 (58.9) |  |
| East North Central | 2169 (15.1) | 835 (32.1) | 1334 (67.9) |  |
| West North Central | 1466 (3.8) | 613 (37.7) | 853 (62.3) |  |
| South Atlantic | 8158 (35.3) | 3544 (37.1) | 4614 (62.9) |  |
| East South Central | 3296 (9.7) | 1366 (34.6) | 1930 (65.4) |  |
| West South Central | 2097 (13.8) | 923 (36.9) | 1174 (63.1) |  |
| Mountain | 530 (2.4) | 220 (36.8) | 310 (63.2) |  |
| Pacific | 708 (7.3) | 269 (36.4) | 439 (64.6) |  |
| **Health Insurance** |  |  |  | **<0.001** |
| Yes | 18419 (87.1) | 8300 (39.1) | 10119 (60.9) |  |
| No | 2136 (13.0) | 457 (19.2) | 1679 (80.8) |  |
| **Primary Care Doctor** |  |  |  | **<0.001** |
| Yes | 17322 (78.8) | 8040 (41.3) | 9282 (58.7) |  |
| No | 3233 (21.2) | 717 (18.9) | 2516 (81.2) |  |
| **Delayed Care due to Cost** |  |  |  | **<0.001** |
| No | 17741 (85.3) | 7849 (38.1) | 9892 (62.0) |  |
| Yes | 2814 (14.8) | 908 (28.0) | 1906 (72.0) |  |
| **Asthma** |  |  |  | **0.004** |
| No | 17205 (83.0) | 7164 (35.8) | 10041 (64.2) |  |
| Yes | 3350 (17.0) | 1593 (40.5) | 1757 (59.5) |  |
| **CVD** |  |  |  | **<0.001** |
| No | 17977 (90.7) | 7374 (35.3) | 10603 (64.7) |  |
| Yes | 2578 (9.3) | 1383 (49.2) | 1195 (50.8) |  |
| **Hypertension** |  |  |  | **<0.001** |
| No | 9643 (57.5) | 3304 (29.7) | 6339 (70.4) |  |
| Yes | 10912 (42.5) | 5453 (45.9) | 5459 (54.1) |  |
| **BMI** |  |  |  | **0.006** |
| Normal/underweight | 4745 (25.3) | 1853 (33.6) | 2892 (66.4) |  |
| Overweight | 6774 (33.3) | 2908 (36.6) | 3866 (63.5) |  |
| Obese | 9036 (41.4) | 3996 (38.4) | 5040 (61.6) |  |
| **Cancer** |  |  |  | **<0.001** |
| No | 18793 (93.9) | 7755 (35.3) | 11038 (64.7) |  |
| Yes | 1762 (6.1) | 1002 (55.5) | 760 (44.5) |  |
| **Diabetes** |  |  |  | **<0.001** |
| No | 15947 (83.9) | 6140 (33.2) | 9807 (66.8) |  |
| Yes | 4608 (16.1) | 2617 (54.1) | 1991 (45.9) |  |

Notes: Boldface indicates statistical significance *p*<0.05.

Notes: Data represent N (weighted percentage) unless otherwise noted Includes data from the 2019 BRFSS annual survey. Analysis includes Non-Hispanic Black participants who completed the 2019 survey from January 2019-April 2020. Analysis excludes US territories. West North Central division includes: Iowa, Kansas, Minnesota, Missouri, Nebraska, North Dakota, and South Dakota. New England division includes: Connecticut, Maine, Massachusetts, New Hampshire, Rhode Island, and Vermont. Middle Atlantic includes: New Jersey, New York, and Pennsylvania. South Atlantic division includes: Delaware, District of Columbia, Florida, Georgia, Maryland, North Carolina, South Carolina, Virginia, and West Virginia. Mountain division includes: Arizona, Colorado, Idaho, New Mexico, Montana, Utah, Nevada, and Wyoming. Pacific division includes: Alaska, California, Hawaii, Oregon, and Washington. East North Central division includes: Indiana, Illinois, Michigan, Ohio, and Wisconsin. East South Central division includes: Alabama, Kentucky, Mississippi, and Tennessee. West South Central division includes: Arkansas, Louisiana, Oklahoma, and Texas.

| **Supplemental Table 3. Hispanic Descriptive Data by Influenza Vaccination, 2019 BRFSS** | | | | |
| --- | --- | --- | --- | --- |
| **N (weighted %)** | | | | |
| N= 18,896 | **Total** | **Percentage who Received Influenza Vaccine** | **Did not Receive Influenza Vaccine** | **P value** |
| **Age, median (IQR)** | 39 (38, 39) | 43 (41, 44) | 37 (36, 38) | **<0.001** |
| **Sex** |  |  |  | **<0.001** |
| Female | 9377 (46.8) | 3965 (38.0) | 5412 (62.0) |  |
| Male | 9519 (53.2) | 3151 (31.2) | 6367 (68.8) |  |
| **Education** |  |  |  | **<0.001** |
| Less than high school | 4094 (29.1) | 1477 (34.3) | 2617 (65.7) |  |
| High school graduate | 5333 (27.6) | 1744 (29.7) | 3589 (70.3) |  |
| Some college | 4728 (27.3) | 1746 (34.5) | 2982 (65.5) |  |
| College graduate or more | 4741 (16.0) | 2149 (42.3) | 2592 (57.7) |  |
| **Income** |  |  |  | 0.077 |
| < $25,000 | 7240 (38.8) | 2672 (34.4) | 4568 (65) |  |
| $25 – $49,999 | 5137 (26.6) | 1749 (32.0) | 3388 (68.0) |  |
| $50 – $74,999 | 2361 (11.9) | 889 (34.9) | 1472 (65.1) |  |
| ≥ $75,000 | 4158 (22.7) | 1806 (36.9) | 2352 (63.1) |  |
| **Divisions** |  |  |  | **<0.001** |
| New England | 1158 (2.4) | 493 (41.3) | 665 (58.7) |  |
| Middle Atlantic | 1073 (7.6) | 442 (39.2) | 631 (60.8) |  |
| East North Central | 1264 (7.8) | 444 (31.2) | 820 (68.8) |  |
| West North Central | 1898 (2.2) | 720 (35.0) | 1178 (65.0) |  |
| South Atlantic | 2575 (14.7) | 908 (30.9) | 1667 (69.1) |  |
| East South Central | 220 (1.2) | 99 (39.8) | 121 (60.2) |  |
| West South Central | 2383 (21.1) | 918 (36.0) | 1465 (64.0) |  |
| Mountain | 4378 (10.7) | 1686 (33.3) | 2692 (66.7) |  |
| Pacific | 3947 (32.4) | 1406 (34.1) | 2541 (65.9) |  |
| **Health Insurance** |  |  |  | **<0.001** |
| Yes | 14432 (73.6) | 6157 (39.6) | 8275 (60.4) |  |
| No | 4464 (26.4) | 959 (19.8) | 3505 (80.2) |  |
| **Primary Care Doctor** |  |  |  | **<0.001** |
| Yes | 12717 (63.1) | 5755 (42.2) | 6962 (57.8) |  |
| No | 6179 (36.9) | 1361 (20.9) | 4818 (79.1) |  |
| **Delayed Care due to Cost** |  |  |  | **<0.001** |
| No | 15269 (79.8) | 6124 (37.1) | 9145 (62.9) |  |
| Yes | 3627 (20.2) | 992 (23.5) | 2635 (76.5) |  |
| **Asthma** |  |  |  | **<0.001** |
| No | 16179 (86.2) | 5932 (33.3) | 10247 (66.7) |  |
| Yes | 2717 (13.9) | 1184 (41.2) | 1533 (58.8) |  |
| **CVD** |  |  |  | **<0.001** |
| No | 17681 (94.7) | 6513 (33.6) | 11168 (66.41) |  |
| Yes | 1215 (5.3) | 603 (48.3) | 612 (51.66 |  |
| **Hypertension** |  |  |  | **<0.001** |
| No | 13673 (76.2) | 4652 (31.4) | 9021 (68.6) |  |
| Yes | 5223 (23.9) | 2464 (43.9) | 2759 (56.1) |  |
| **BMI** |  |  |  | **0.035** |
| Normal/underweight | 5243 (27.5) | 1878 (31.7) | 3365 (68.3) |  |
| Overweight | 7050 (37.6) | 2660 (35.3) | 4390 (64.7) |  |
| Obese | 6603 (34.8) | 2578 (35.4) | 4025 (64.6) |  |
| **Cancer** |  |  |  | **<0.001** |
| No | 17763 (94.9) | 6511 (33.6) | 11252 (66.4) |  |
| Yes | 1133 (5.1) | 605 (48.6) | 528 (51.4) |  |
| **Diabetes** |  |  |  | **<0.001** |
| No | 16192 (87.7) | 5695 (32.4) | 10497 (67.7) |  |
| Yes | 2704 (12.3) | 1421 (48.7) | 1283 (51.3) |  |

Notes: Boldface indicates statistical significance *p*<0.05.

Notes: Data represent N (weighted percentage) unless otherwise noted Includes data from the 2019 BRFSS annual survey. Analysis includes Hispanic participants who completed the 2019 survey from January 2019-April 2020. Analysis excludes US territories. West North Central division includes: Iowa, Kansas, Minnesota, Missouri, Nebraska, North Dakota, and South Dakota. New England division includes: Connecticut, Maine, Massachusetts, New Hampshire, Rhode Island, and Vermont. Middle Atlantic includes: New Jersey, New York, and Pennsylvania. South Atlantic division includes: Delaware, District of Columbia, Florida, Georgia, Maryland, North Carolina, South Carolina, Virginia, and West Virginia. Mountain division includes: Arizona, Colorado, Idaho, New Mexico, Montana, Utah, Nevada, and Wyoming. Pacific division includes: Alaska, California, Hawaii, Oregon, and Washington. East North Central division includes: Indiana, Illinois, Michigan, Ohio, and Wisconsin. East South Central division includes: Alabama, Kentucky, Mississippi, and Tennessee. West South Central division includes: Arkansas, Louisiana, Oklahoma, and Texas.

| **Supplemental Table 4. Asian Descriptive Data by Influenza Vaccination, 2019 BRFSS** | | | | |
| --- | --- | --- | --- | --- |
| **N (weighted %)** | | | | |
| N= 5,516 | **Total** | **Percentage who Received Influenza Vaccine** | **Did not Receive Influenza Vaccine** | **P value** |
| **Age, median (IQR)** | 38 (37, 40) | 42 (40, 44) | 35 (33, 37) | **<0.001** |
| **Sex** |  |  |  | 0.136 |
| Female | 2456 (47.6) | 1238 (46.1) | 1218 (53.9) |  |
| Male | 3060 (52.4) | 1356 (42.3) | 1704 (57.7) |  |
| **Education** |  |  |  | **0.001** |
| Less than high school | 133 (3.9) | 58 (52.6) | 75 (47.4) |  |
| High school graduate | 843 (15.9) | 340 (36.1) | 503 (63.9) |  |
| Some college | 1161 (23.6) | 507 (38.7) | 654 (61.4) |  |
| College graduate or more | 3379 (56.6) | 1689 (48.0) | 1690 (52.0) |  |
| **Income** |  |  |  | **0.004** |
| < $25,000 | 987 (19.2) | 403 (40.6) | 584 (59.4) |  |
| $25 – $49,999 | 1149 (18.9) | 484 (36.9) | 665 (63.1) |  |
| $50 – $74,999 | 846 (12.9) | 384 (42.4) | 462 (57.6) |  |
| ≥ $75,000 | 2534 (48.9) | 1323 (48.7) | 1211 (51.3) |  |
| **Divisions** |  |  |  | 0.600 |
| New England | 376 (3.5) | 173 (44.4) | 203 (55.6) |  |
| Middle Atlantic | 378 (11.6) | 170 (40.8) | 208 (59.2) |  |
| East North Central | 380 (9.7) | 165 (44.7) | 215 (55.3) |  |
| West North Central | 432 (2.7) | 186 (40.6) | 246 (59.4) |  |
| South Atlantic | 664 (12.1) | 336 (43.0) | 328 (57.0) |  |
| East South Central | 67 (0.8) | 27 (33.7) | 40 (66.3) |  |
| West South Central | 238 (8.5) | 104 (39.6) | 134 (60.4) |  |
| Mountain | 346 (4.1) | 159 (40.2) | 187 (59.8) |  |
| Pacific | 2635 (47.0) | 1274 (46.6) | 1361 (53.4) |  |
| **Health Insurance** |  |  |  | **<0.001** |
| Yes | 5079 (90.1) | 2490 (46.1) | 2589 (53.9) |  |
| No | 437 (10.0) | 104 (25.9) | 333 (74.1) |  |
| **Primary Care Doctor** |  |  |  | **<0.001** |
| Yes | 4351 (76.9) | 2269 (48.9) | 2082 (51.1) |  |
| No | 1165 (23.2) | 325 (28.1) | 840 (72.0) |  |
| **Delayed Care due to Cost** |  |  |  | **0.004** |
| No | 5058 (90.9) | 2429 (45.2) | 2629 (54.8) |  |
| Yes | 458 (9.1) | 165 (33.2) | 293 (66.8) |  |
| **Asthma** |  |  |  | 0.522 |
| No | 4938 (90.1) | 2286 (43.8) | 2652 (56.2) |  |
| Yes | 578 (10.0) | 308 (46.7) | 270 (53.3) |  |
| **CVD** |  |  |  | **0.002** |
| No | 5223 (95.1) | 2419 (43.0) | 2804 (57.0) |  |
| Yes | 293 (4.9) | 175 (64.4) | 118 (35.6) |  |
| **Hypertension** |  |  |  | **<0.001** |
| No | 4033 (80.4) | 1747 (41.3) | 2286 (58.7) |  |
| Yes | 1483 (19.6) | 847 (55.6) | 636 (44.4) |  |
| **BMI** |  |  |  | 0.431 |
| Normal and underweight | 2939 (55.1) | 1364 (44.2) | 1575 (55.8) |  |
| Overweight | 1856 (33.6) | 873 (42.5) | 983 (57.5) |  |
| Obese | 721 (11.3) | 357 (48.3) | 364 (51.7) |  |
| **Cancer** |  |  |  | 0.060 |
| No | 5238 (96.9) | 2416 (43.6) | 2822 (56.4) |  |
| Yes | 278 (3.1) | 178 (58.6) | 100 (41.4) |  |
| **Diabetes** |  |  |  | **<0.001** |
| No | 4898 (92.3) | 2208 (42.9) | 2690 (57.1) |  |
| Yes | 618 (7.7) | 386 (58.8) | 232 (41.2) |  |

Notes: Boldface indicates statistical significance *p*<0.05.

Notes: Data represent N (weighted percentage) unless otherwise noted Includes data from the 2019 BRFSS annual survey. Analysis includes Asian participants who completed the 2019 survey from January 2019-April 2020. Analysis excludes US territories. West North Central division includes: Iowa, Kansas, Minnesota, Missouri, Nebraska, North Dakota, and South Dakota. New England division includes: Connecticut, Maine, Massachusetts, New Hampshire, Rhode Island, and Vermont. Middle Atlantic includes: New Jersey, New York, and Pennsylvania. South Atlantic division includes: Delaware, District of Columbia, Florida, Georgia, Maryland, North Carolina, South Carolina, Virginia, and West Virginia. Mountain division includes: Arizona, Colorado, Idaho, New Mexico, Montana, Utah, Nevada, and Wyoming. Pacific division includes: Alaska, California, Hawaii, Oregon, and Washington. East North Central division includes: Indiana, Illinois, Michigan, Ohio, and Wisconsin. East South Central division includes: Alabama, Kentucky, Mississippi, and Tennessee. West South Central division includes: Arkansas, Louisiana, Oklahoma, and Texas.

| **Supplemental Table 5. American Indian / Alaska Native Descriptive Data by Influenza Vaccination, 2019 BRFSS** | | | | |
| --- | --- | --- | --- | --- |
| **N (weighted %)** | | | | |
| N= 4,439 | **Total** | **Percentage who Received Influenza Vaccine** | **Did not Receive Influenza Vaccine** | **P value** |
| **Age, median (IQR)** | 48 (46, 49) | 54 (52, 57) | 45 (43, 47) | **<0.001** |
| **Sex** |  |  |  | **<0.001** |
| Female | 2364 (47.8) | 1110 (42.3) | 1254 (57.7) |  |
| Male | 2075 (52.2) | 820 (31.2) | 1255 (68.8) |  |
| **Education** |  |  |  | **0.003** |
| Less than high school | 548 (16.9) | 206 (38.1) | 342 (61.9) |  |
| High school graduate | 1397 (33.7) | 536 (33.3) | 861 (66.7) |  |
| Some college | 1415 (32.9) | 617 (33.7) | 798 (66.3) |  |
| College graduate or more | 1079 (16.5) | 517 (47.0) | 508 (53.0) |  |
| **Income** |  |  |  | 0.810 |
| < $25,000 | 2030 (39.5) | 836 (37.7) | 1194 (62.3) |  |
| $25 – $49,999 | 1110 (24.7) | 480 (35.0) | 630 (65.0) |  |
| $50 – $74,999 | 542 (14.0) | 249 (38.7) | 293 (61.3) |  |
| ≥ $75,000 | 757 (21.9) | 365 (34.7) | 392 (65.3) |  |
| **Divisions** |  |  |  | **0.045** |
| New England | 158 (2.4) | 61 (30.6) | 97 (69.4) |  |
| Middle Atlantic | 89 (6.7) | 38 (41.5) | 51 (58.6) |  |
| East North Central | 225 (9.6) | 81 (28.8) | 144 (71.2) |  |
| West North Central | 1153 (9.8) | 550 (38.7) | 603 (61.3) |  |
| South Atlantic | 420 (13.5) | 148 (31.5) | 272 (68.6) |  |
| East South Central | 189 (6.8) | 68 (28.0) | 121 (72.0) |  |
| West South Central | 445 (18.0) | 194 (37.4) | 251 (62.6) |  |
| Mountain | 1342 (19.6) | 615 (43.0) | 727 (57.0) |  |
| Pacific | 418 (13.7) | 175 (37.8) | 243 (62.2) |  |
| **Health Insurance** |  |  |  | **<0.001** |
| Yes | 4039 (88.9) | 1844 (39.1) | 2195 (60.9) |  |
| No | 400 (11.1) | 86 (15.5) | 314 (84.5) |  |
| **Primary Care Doctor** |  |  |  | **<0.001** |
| Yes | 3211 (73.9) | 1569 (41.6) | 1642 (58.4) |  |
| No | 1228 (26.1) | 361 (22.1) | 867 (78.0) |  |
| **Delayed Care due to Cost** |  |  |  | **0.011** |
| No | 3755 (82.5) | 1694 (38.2) | 2061 (61.8) |  |
| Yes | 684 (17.5) | 236 (28.6) | 448 (71.4) |  |
| **Asthma** |  |  |  | **0.032** |
| No | 3643 (78.4) | 1571 (34.0) | 2072 (66.0) |  |
| Yes | 796 (21.6) | 359 (45.6) | 437 (54.4) |  |
| **CVD** |  |  |  | **0.004** |
| No | 3700 (85.1) | 1547 (34.5) | 2153 (65.6) |  |
| Yes | 739 (14.9) | 383 (48.3) | 356 (51.7) |  |
| **Hypertension** |  |  |  | **<0.001** |
| No | 2492 (62.1) | 943 (31.1) | 1549 (68.9) |  |
| Yes | 1947 (37.9) | 987 (45.4) | 960 (54.7) |  |
| **BMI** |  |  |  | 0.173 |
| Normal/underweight | 1061 (26.4) | 437 (37.1) | 624 (62.9) |  |
| Overweight | 1482 (35.3) | 636 (32.7) | 846 (67.3) |  |
| Obese | 1896 (38.4) | 857 (39.6) | 1039 (60.4) |  |
| **Cancer** |  |  |  | 0.133 |
| No | 3903 (87.3) | 1659 (35.2) | 2244 (64.8) |  |
| Yes | 536 (12.7) | 271 (45.6) | 265 (54.4) |  |
| **Diabetes** |  |  |  | **<0.001** |
| No | 3438 (82.0) | 1381 (33.5) | 2057 (66.5) |  |
| Yes | 1001 (18.0) | 549 (50.4) | 452 (49.6) |  |

Notes: Boldface indicates statistical significance *p*<0.05.

Notes: Data represent N (weighted percentage) unless otherwise noted Includes data from the 2019 BRFSS annual survey. Analysis includes American Indian / Alaska Native participants who completed the 2019 survey from January 2019-April 2020. Analysis excludes US territories. West North Central division includes: Iowa, Kansas, Minnesota, Missouri, Nebraska, North Dakota, and South Dakota. New England division includes: Connecticut, Maine, Massachusetts, New Hampshire, Rhode Island, and Vermont. Middle Atlantic includes: New Jersey, New York, and Pennsylvania. South Atlantic division includes: Delaware, District of Columbia, Florida, Georgia, Maryland, North Carolina, South Carolina, Virginia, and West Virginia. Mountain division includes: Arizona, Colorado, Idaho, New Mexico, Montana, Utah, Nevada, and Wyoming. Pacific division includes: Alaska, California, Hawaii, Oregon, and Washington. East North Central division includes: Indiana, Illinois, Michigan, Ohio, and Wisconsin. East South Central division includes: Alabama, Kentucky, Mississippi, and Tennessee. West South Central division includes: Arkansas, Louisiana, Oklahoma, and Texas.

| **Supplemental Table 6. Native Hawaiian or Other Pacific Islander Descriptive Data by Influenza Vaccination, 2019 BRFSS** | | | | |
| --- | --- | --- | --- | --- |
| **N (weighted %)** | | | | |
| N=957 | **Total** | **Percentage who Received Influenza Vaccine** | **Did not Receive Influenza Vaccine** | **P value** |
| **Age, median (IQR)** | 39 (36, 42) | 40 (35, 47) | 39 (35, 42) | **<0.001** |
| **Sex** |  |  |  | **0.036** |
| Female | 495 (49.0) | 225 (44.1) | 270 (55.9) |  |
| Male | 462 (51.0) | 142 (30.5) | 320 (69.6) |  |
| **Education** |  |  |  | 0.407 |
| Less than high school | 47 (7.3) | 19 (22.1) | 28 (77.9) |  |
| High school graduate | 376 (36.9) | 137 (38.3) | 239 (61.7) |  |
| Some college | 283 (33.3) | 105 (34.8) | 178 (65.2) |  |
| College graduate or more | 251 (22.5) | 106 (43.5) | 145 (56.5) |  |
| **Income** |  |  |  | 0.854 |
| < $25,000 | 280 (26.6) | 100 (34.1) | 180 (65.9) |  |
| $25 – $49,999 | 250 (22.8) | 98 (35.0) | 152 (65.1) |  |
| $50 – $74,999 | 143 (14.4) | 53 (45.5) | 90 (54.5) |  |
| ≥ $75,000 | 284 (36.2) | 116 (37.4) | 168 (62.6) |  |
| **Divisions** |  |  |  | 0.492 |
| New England | 25 (1.6) | 14 (39.1) | 11 (62.9) |  |
| Middle Atlantic | 34 (9.2) | 11 (27.3) | 23 (72.7) |  |
| East North Central | 19 (5.6) | 5 (31.1) | 14 (68.9) |  |
| West North Central | 37 (4.6) | 16 (55.5) | 21 (44.5) |  |
| South Atlantic | 59 (10.8) | 26 (43.8) | 33 (56.2) |  |
| East South Central | 17 (2.3) | 6 (22.7) | 11 (77.4) |  |
| West South Central | 25 (5.2) | 11 (42.0) | 14 (58.0) |  |
| Mountain | 77 (11.6) | 26 (22.9) | 51 (77.1) |  |
| Pacific | 664 (49.1) | 252 (40.0) | 412 (60.0) |  |
| **Health Insurance** |  |  |  | **<0.001** |
| Yes | 846 (87.6) | 346 (40.3) | 500 (59.7) |  |
| No | 111 (12.4) | 21 (14.6) | 90 (85.4) |  |
| **Primary Care Doctor** |  |  |  | **<0.001** |
| Yes | 765 (77.5) | 332 (44.0) | 433 (56.0) |  |
| No | 192 (22.5) | 35 (13.5) | 157 (86.5) |  |
| **Delayed Care due to Cost** |  |  |  | 0.358 |
| No | 833 (88.7) | 329 (38.0) | 504 (62.0) |  |
| Yes | 124 (11.4) | 38 (30.5) | 86 (69.5) |  |
| **Asthma** |  |  |  | 0.926 |
| No | 765 (80.0) | 291 (37.0) | 474 (63.0) |  |
| Yes | 192 (20.0) | 76 (37.7) | 116 (62.3) |  |
| **CVD** |  |  |  | **0.040** |
| No | 867 (92.4) | 316 (35.7) | 551 (64.3) |  |
| Yes | 90 (7.6) | 51 (54.9) | 39 (45.1) |  |
| **Hypertension** |  |  |  | 0.051 |
| No | 624 (74.6) | 206 (34.0) | 418 (66.1) |  |
| Yes | 333 (25.4) | 161 (46.5) | 172 (53.5) |  |
| **BMI** |  |  |  | 0.397 |
| Normal/underweight | 227 (24.0) | 88 (38.1) | 139 (61.9) |  |
| Overweight | 300 (32.3) | 113 (31.0) | 187 (69) |  |
| Obese | 430 (43.7) | 166 (41.1) | 264 (58.9) |  |
| **Cancer** |  |  |  | 0.254 |
| No | 893 (94.4) | 335 (36.2) | 558 (63.8) |  |
| Yes | 64 (5.6) | 32 (53.7) | 32 (46.3) |  |
| **Diabetes** |  |  |  | 0.475 |
| No | 805 (88.6) | 289 (36.5) | 516 (63.5) |  |
| Yes | 152 (11.4) | 78 (42.4) | 74 (57.6) |  |

Notes: Boldface indicates statistical significance *p*<0.05.

Notes: Data represent N (weighted percentage) unless otherwise noted Includes data from the 2019 BRFSS annual survey. Analysis includes Native Hawaiian or Other Pacific Islander participants who completed the 2019 survey from January 2019-April 2020. Analysis excludes US territories. West North Central division includes: Iowa, Kansas, Minnesota, Missouri, Nebraska, North Dakota, and South Dakota. New England division includes: Connecticut, Maine, Massachusetts, New Hampshire, Rhode Island, and Vermont. Middle Atlantic includes: New Jersey, New York, and Pennsylvania. South Atlantic division includes: Delaware, District of Columbia, Florida, Georgia, Maryland, North Carolina, South Carolina, Virginia, and West Virginia. Mountain division includes: Arizona, Colorado, Idaho, New Mexico, Montana, Utah, Nevada, and Wyoming. Pacific division includes: Alaska, California, Hawaii, Oregon, and Washington. East North Central division includes: Indiana, Illinois, Michigan, Ohio, and Wisconsin. East South Central division includes: Alabama, Kentucky, Mississippi, and Tennessee. West South Central division includes: Arkansas, Louisiana, Oklahoma, and Texas.
